# The c-MET receptor tyrosine kinase contributes to neutrophil-driven pathology in cutaneous leishmaniasis

**DOI:** 10.1371/journal.ppat.1010247

**Published:** 2022-01-18

**Authors:** Katiuska Passelli, Borja Prat-Luri, Margot Merlot, Michiel Goris, Massimiliano Mazzone, Fabienne Tacchini-Cottier

**Affiliations:** 1 Department of Biochemistry, Faculty of Biology and Medicine, University of Lausanne, Epalinges, Switzerland; 2 World Health Organization Collaborating Centre for Immunology Research and Training, Faculty of Biology and Medicine, University of Lausanne, Epalinges, Switzerland; 3 Laboratory of Molecular Oncology and Angiogenesis, Vesalius Research Center, Department of Oncology, KU Leuven, Leuven, Belgium; 4 Laboratory of Molecular Oncology and Angiogenesis, Vesalius Research Center, VIB, Leuven, Belgium; INRS - Institut Armand Frappier, CANADA

## Abstract

Neutrophils are the first line of defence against invading pathogens. Although neutrophils are well-known professional killers, some pathogens including *Leishmania (L*.*) parasites* survive in neutrophils, using these cells to establish infection. Manipulation of neutrophil recruitment to the infection site is therefore of interest in this cutaneous disease. The c-MET tyrosine kinase receptor was shown to promote neutrophil migration to inflamed sites. Here, we investigated the importance of c-MET expression on neutrophils in their recruitment to the infection site and the role of c-Met expression in the pathology of leishmaniasis. Following infection with *L*. *mexicana*, mice with conditional deletion of c-MET in neutrophils controlled significantly better their lesion development and parasite burden compared to similarly infected wild type mice. Our data reveal a specific role for c-MET activation in *Leishmania*-induced neutrophil infiltration, a process correlating with their negative role in the pathology of the diseases. We further show that c-MET phosphorylation is observed in established cutaneous lesions. Exposure to *L*. *mexicana* upregulated c-Met expression predominantly in infected neutrophils and c-Met expression influenced ROS release by neutrophils. In addition, pharmacological inhibition of c-MET, administrated once the lesion is established, induced a significant decrease in lesion size associated with diminished infiltration of neutrophils. Both genetic ablation of c-MET in neutrophils and systemic inhibition of c-MET locally resulted in higher levels of CD4^+^T cells producing IFNγ, suggesting a crosstalk between neutrophils and these cells. Collectively, our data show that c-MET activation in neutrophils contributes to their recruitment following infection, and that *L*. *mexicana* induction of c-MET on neutrophils impacts the local pathology associated with this disease. Our results suggest a potential use for this inhibitor in the control of the cutaneous lesion during this parasitic infection.

## Author summary

The leishmaniases are a complex of diseases caused by *Leishmania* protozoan parasites. There exist different forms of the disease, ranging from cutaneous lesions to visceral disease, the latter being fatal if not treated. Cutaneous leishmaniasis is the most common form with one million new cases occurring annually. Increasing evidence points to an important role for neutrophils in disease outcome and these cells were shown to play a deleterious role following infection with several *Leishmania* spp., including *L*. *mexicana*. Here, we investigated the role of the c-MET receptor tyrosine kinase in the outcome of cutaneous leishmaniasis. c-MET has been shown to be involved in neutrophil recruitment to inflamed sites. We show here that *L*. *mexicana* induce c-MET expression and phosphorylation predominantly in neutrophils. Using mice genetically deficient for c-MET in neutrophils, a better control of lesion size and parasite load was observed. Furthermore, injection of a specific inhibitor of c-MET, once the lesion is established, significantly decreased the cutaneous pathology. The results presented here reveal that c-MET is contributing to the pathology induced by *L*. *mexicana* and suggest that the use of c-MET inhibitors could ameliorate the cutaneous lesion pathology.

## Introduction

c-MET is a tyrosine kinase encoded by the c-MET proto-oncogene, it is the only known receptor for the hepatocyte growth factor (HGF). HGF/c-MET signalling regulates numerous biological pathways such as proliferation, survival, migration and tissue regeneration [1]. Aberrant activation of c-MET signalling has been shown to participate in tumour progression [2–5], prompting the development of c-MET inhibitors. However, c-MET inhibitors showed only limited success in clinical trials [6]. Notably, this restricted efficacy of systemic c-MET inhibition in cancer is in part related to the inhibition of c-MET in neutrophils. Indeed, c-MET was shown to be crucial for the recruitment of anti-tumoral neutrophils, that kill cancer cells following nitric oxide production [7].

In addition to the role of neutrophils in cancer, neutrophils are also the first line of defence against invading pathogens. However, in addition to their well-characterized killing functions, neutrophils can also play deleterious roles in several infections including some forms of leishmaniasis [8,9].

*Leishmania* (*L*.) are obligate intracellular protozoan parasites that cause the leishmaniases, a spectrum of vector-borne infectious diseases ranging from cutaneous to visceral forms. During their blood meals, female sandfly vectors deposit metacyclic promastigotes in the host skin. At the site of infection, *Leishmania* are first internalized within hours by recruited neutrophils and then transferred to macrophages where they differentiate into the replicative non-flagellated amastigote form [10,11]. *L*. *mexicana* is a New World *Leishmania* species, causing chronic unhealing lesions in humans and mice. The severity of the disease correlates with the limited recruitment of monocytes and poor development of a Th1 immune response [12–14]. We previously showed that the early neutrophil recruitment to the infection site plays a detrimental role during *L*. *mexicana* infection, as in contrast to wild type mice, neutropenic mice showed an early increase in monocytes and dendritic cells recruitment inducing the subsequent development of a protective Th1 immune response and mice were able to heal their lesion and better control their parasite burden [8].

*L*. *major* was shown to enter neutrophils and persist in these cells, a process referred to as the Trojan horse model [15,16]. Persistence of live parasites in neutrophils was also observed following *L*. *mexicana* infection [8,17]. Trapping without killing of *Leishmania* in neutrophils is thought to promote subsequent parasite dissemination into the host.

Here, we aimed to investigate the role of c-MET activation in neutrophils in the context of parasite infection, more specifically following infection with *Leishmania mexicana*. We report here a prominent role for c-MET expression on neutrophils in the control of *Leishmania*-induced cutaneous pathology.

## Results

### *Leishmania* parasites induce c-MET mRNA expression in neutrophils

**Fig 1:**
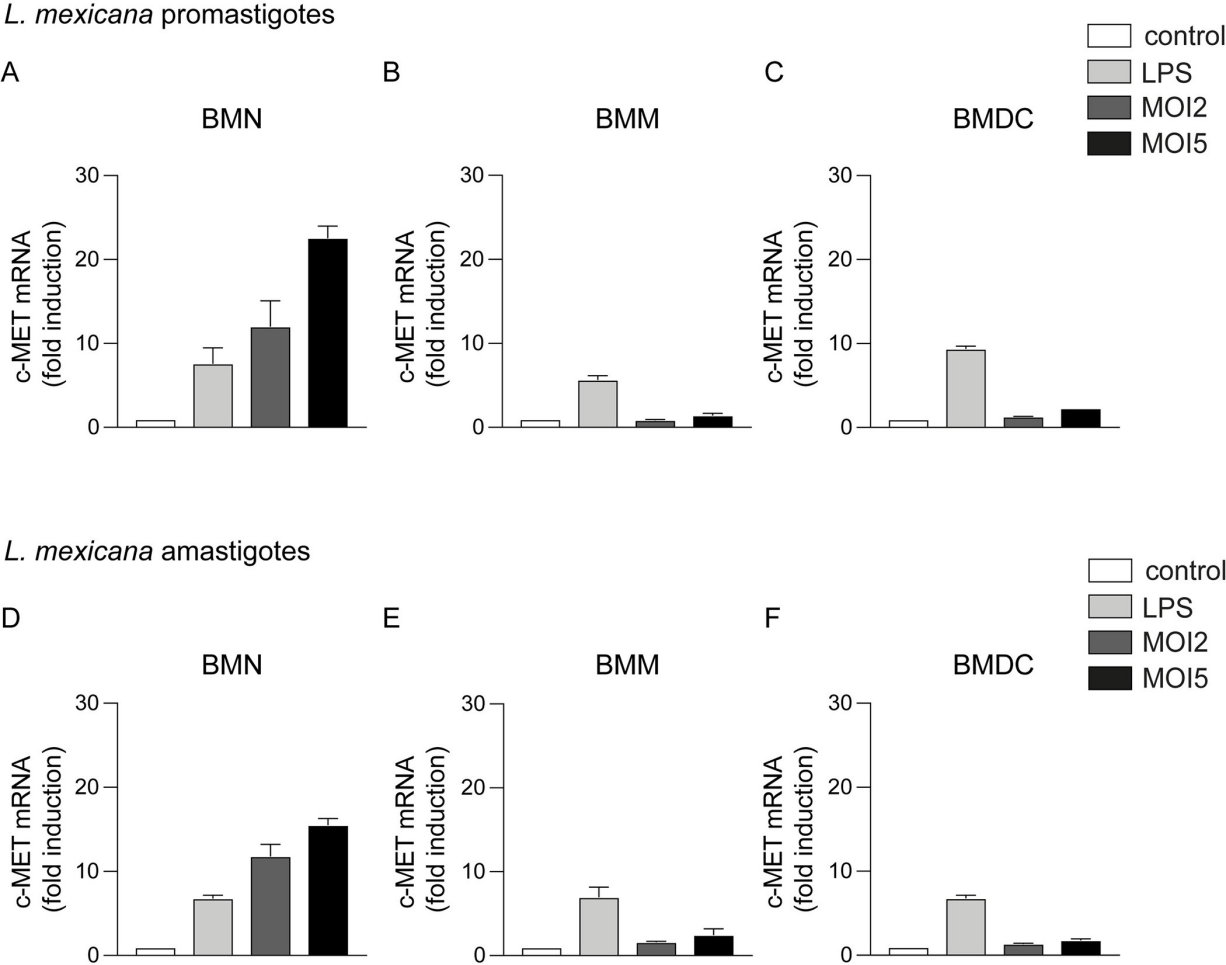
*Leishmania* parasites upregulate c-MET mRNA expression in neutrophils. **A**) BM isolated neutrophils (BMNs) were co-cultured at the indicated multiplicity of infection (MOI) with the metacyclic (infecting) form of the *L*. *mexicana* parasites as indicated. 16h later mRNA was extracted, and c-MET mRNA levels analysed by RT-qPCR. Uninfected cells were used as negative control and cells treated with 50 ng/mL of LPS were used as positive control. **B**) BM-derived macrophages (BMMs) and **C**) BM-derived dendritic cells (BMDCs) were processed similarly. **D-F**) The indicated bone marrow-derived myeloid cells were similarly infected with amastigotes, the replicating form of the parasites. The data are normalized to endogenous levels of HPRT mRNA and expressed as fold increase relative to expression levels detected in control cells. The data are representative of ≥ 3 independent experiments, n = 3/group.

In order to investigate the role of c-MET in neutrophils during *L*. *mexicana* infection, we first assessed by qPCR whether the parasite could induce *c-MET* mRNA expression *in vitro*. To this end, bone marrow neutrophils (BMNs) were isolated from the femur of C57BL/6 mice and the cells were exposed for 16h to *L*. *mexicana* metacyclic promastigotes, the infective form of the parasite, at a multiplicity of infection (MOI) of 2 and 5. As positive control, neutrophils were treated for 16h with LPS. Induction of c-MET mRNA in neutrophils was increased ten to twenty-fold at MOI of 2 and 5, respectively, compared to the mRNA levels observed in non-exposed neutrophils (**Fig 1A**). To assess if infection impacted survival of neutrophils *in vitro*, BMNs were exposed to *L*. *mexicana* at the indicated MOIs and 16 hours post infection, neutrophils were stained with AnnexinV and DAPI and their apoptotic status analysed by flow cytometry. Exposure to *L*. *mexicana* did not impact the survival (AnnexinV^-^DAPI^-^) nor the apoptotic status (AnnexinV^+^DAPI^+/-^) of neutrophils *in vit*ro (**S1A Fig**). These data demonstrate that the increase in c-MET mRNA observed in infected neutrophils is not due to differences in neutrophil survival between uninfected and infected neutrophils. We further analysed whether *L*. *mexicana* promotes c-MET expression in other myeloid cells participating in the innate immune response against the parasite, such as macrophages and dendritic cells. Bone marrow-derived macrophages (BMMs) were incubated *in vitro* for 16h with *L*. *mexicana* promastigotes. Compared to non-exposed macrophages, no difference (MOI of 2) or only a slight increase (MOI of 5) in c-MET mRNA expression was observed following incubation with *Leishmania* promastigotes (**Fig 1B**). Similarly, *L*. *mexicana* promastigotes induced only a slight increase in c-MET expression in bone marrow-derived dendritic cells (BMDCs) (**Fig 1C**). We then investigated whether amastigotes, the replicative form of the parasite which is present during the chronic phase of the disease, was also able to induce c-MET mRNA expression in neutrophils. BMNs, BMMs and BMDCs were co-cultured with amastigotes for 16 hours, and c-MET mRNA was measured. Amastigotes induced c-MET mRNA, with levels up to ten-fold at MOI2 and fifteen-fold at MOI5 higher than those observed in uninfected (control) neutrophils (**Fig 1D**). Very low mRNA induction was observed in BMMs and BMDCs exposed to *L*. *mexicana* amastigotes (**Fig 1E and 1F**, respectively). These findings indicate that *L*. *mexicana* promotes a strong dose-dependent upregulation of c-MET mRNA expression specifically in neutrophils. c-MET mRNA levels were also selectively upregulated in neutrophils following exposure to *L*. *major* LV39 **(S2 Fig)**, showing that the upregulation of c-MET on neutrophils by *Leishmania mexicana* extends to other *Leishmania* spp.

### *Leishmania mexicana* induce c-MET expression selectively on parasitized neutrophils

**Fig 2:**
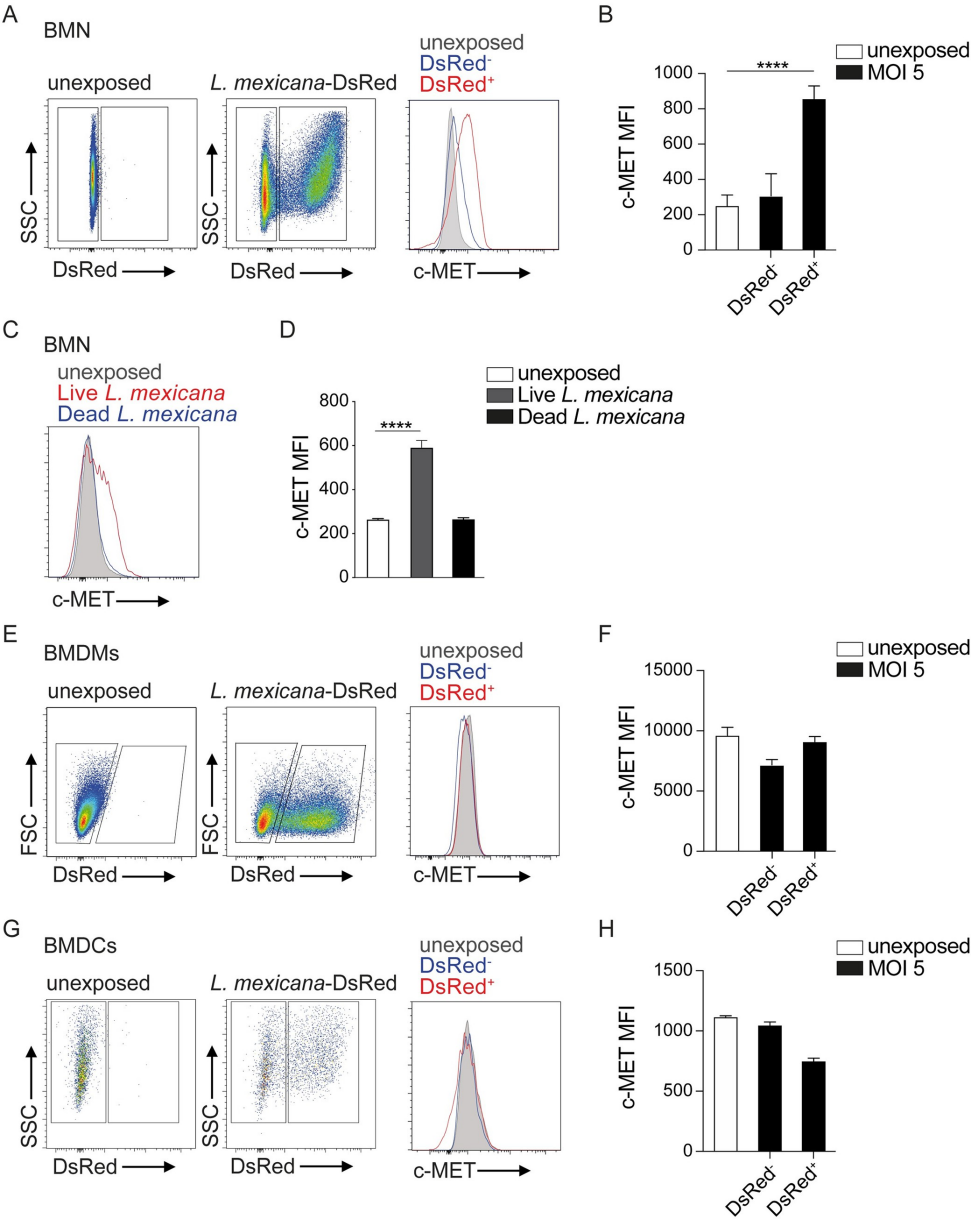
*Leishmania mexicana* upregulates c-MET expression on neutrophil surface. **A**). BM PMNs were isolated and co-cultured *in vitro* in presence or absence (unexposed) of *L*. *mexicana*-DsRed metacyclic promastigotes at a MOI of 5 for 16h. **A**) Representative flow cytometry plot showing PMNs unexposed (left) and PMNs exposed to parasites that are either infected (*L*. *mexicana*-DsRed^+^) or not (*L*. *mexicana*-DsRed^-^). c-MET expression in infected or non-infected neutrophils is shown on the histogram and **B**) the corresponding MFI of a representative experiment performed at the indicated MOI is given. **C**) BMNs were isolated and exposed to live or dead *L*. *mexicana* parasites for 16h at a MOI of 5 and the equivalent for dead parasites. c-MET expression is shown on a representative histogram and **D**) the corresponding MFI is given. **E**) BMMs were co-cultured with *L*. *mexicana*-DsRed at MOI of 5 for 16h. c-MET expression was analysed as indicated in **A**. A representative flow cytometry plot, histogram and **F**) the corresponding MFI of a representative experiment are shown. **G**) BMDCs were co-cultured with *L*. *mexicana*-DsRed and c-MET expression was analysed by flow cytometry. Representative flow cytometry plots, the related histogram and **H**) the corresponding MFI of a representative experiment are shown. Data are representative of ≥ 2 independent experiments, n≥3.

To further explore the impact of infection on c-MET expression, we determined whether parasite internalisation induced c-MET surface expression in neutrophils harboring or not parasites. First, BMNs were exposed for 16h to fluorescent *L*. *mexicana*-DsRed at MOI of 5 and c-MET expression was assessed by flow cytometry. Non-infected (unexposed) neutrophils were used as a control. A significant increase of c-MET expression was observed in infected neutrophils (DsRed^+^) compared to non-infected cells (DsRed^-^), as shown in the representative flow cytometry plot (**Fig 2A),** and the corresponding mean fluorescent intensity (MFI) **(Fig 2B**). We further show that parasites need to be alive to induce c-MET expression in neutrophils, as demonstrated by the lack of c-MET induction observed in response to dead *Leishmania* parasites (**Fig 2C and 2D**). To investigate if *Leishmania* triggering of TLR receptors is involved in c-MET induction on neutrophils, we exposed MyD88^-/-^, TLR4^-/-^ and WT neutrophils to *L*. *mexicana* and analyzed the levels of c-MET expression by flow cytometry. No difference in c-MET induction was observed in all the conditions tested, showing that *L*. *mexicana*-dependent c-MET induction in neutrophils is TLR-independent **(S3A and S3B Fig)**. To assess if c-MET induction depended on nuclear factor (NF)-κΒ activation, BMNs were treated or not with the highly selective IκΒ (IKK) inhibitor, which is critical for the activation of NF-κΒ [18], and infected with *L*. *mexicana*. Impairment of NF-κΒ activation resulted in partially decreased *L*. *mexicana-*induced c-MET levels (**S3C Fig**). These data show that c-MET induction by *L*. *mexicana* is TLR independent and involves NF-κB activation.

We also assessed c-MET surface expression in *L*. *mexicana infected* BMMs. Consistent with our mRNA data, infected and non-infected macrophages expressed low levels of c-MET (**Fig 2E and 2F**), and we did not observe any change in c-MET expression between non-infected and infected BMDCs (**Fig 2G and 2H**). Collectively, these data indicate that *in vitro*, c-MET expression is upregulated specifically in neutrophils that have phagocytosed the parasites suggesting that the parasite itself is inducing c-MET expression.

**Fig 3:**
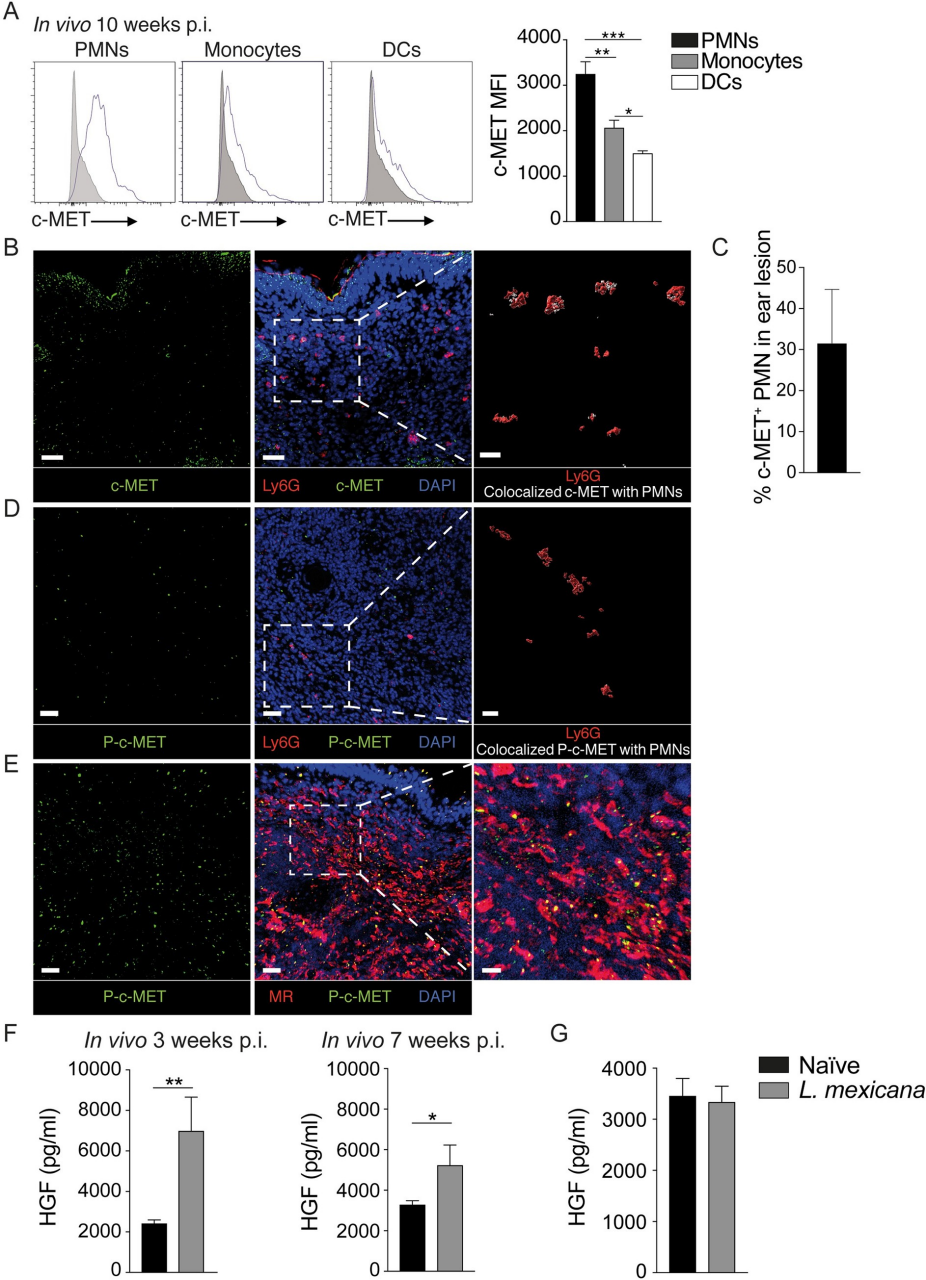
c-MET expression and phosphorylation in infected ears. C57BL/6 mice were infected with 10^6^ *L*. *mexicana*, and seven weeks later, the infected ears were collected, and a cell suspension obtained. **A**) c-MET expression in CD45^+^CD11b^+^Ly6G^+^ PMNs, CD45^+^CD11b^+^Ly6G^-^Ly6C^+^ monocytes and CD45^+^CD11c^+^ DCs was assessed by flow cytometry and c-MET expression shown in the histograms. The corresponding MFI of a representative experiment is shown. Shaded histograms are the fluorescence minus one (FMO) control of cells isolated from similarly infected C57BL/6 mice. **B**) Representative images of sections of *L*. *mexicana* infected ears seven weeks p.i., showing immunofluorescence staining for c-MET (green), Ly6G^+^ neutrophils (red) and DAPI^+^ (blue) nuclei with an enlargement (right) showing a 3D representation of Ly6G (red) and c-MET co-localization (white) in neutrophils. **C**) The quantification of c-MET^+^ neutrophils in the analyzed ear section is shown in the bar graph. **D**) Representative images of ear sections stained for phospho-c-MET, Ly6G^+^ neutrophils and DAPI^+^ nuclei. An enlargement (right) of a 3D representation of Ly6G (red) and phospho-c-MET co-localization (white) in neutrophils is shown. **E**) Section of a representative section of an infected ear stained for phospho-c-MET (left) and mannose receptor and DAPI (right). Scale bars: 30 μm (left). Right: enlargement of the defined area, scale bars: 10 μm. **F**) Three and seven weeks post-infection, the infected ears were collected and homogenized in RIPA buffer using a tissue lyser. The level of HGF in the ear lysate was assessed by ELISA. Non-infected ears were used as control. **G**) The serum from these mice was isolated and analyzed for HGF levels. **A-E** are representative of ≥2 independent experiments, n>3/group. **F-G** is representative of 2 independent experiments n≥5/group. **p <0.01.

We then analysed c-MET expression *in vivo* at the site of infection, during the chronic phase of *L*. *mexicana* disease. C57BL/6 mice were intradermally (i.d.) infected with *L*. *mexicana* metacyclic promastigotes in the ear. Ten weeks after the infection, infected ears were collected, processed and c-MET expression in neutrophils, monocytes and dendritic cells was analysed by flow cytometry. The highest level of c-MET was observed in neutrophils, whereas monocytes and dendritic cells showed significantly lower expression. (**Fig 3A**). The levels of c-MET were also assessed in situ, by immunofluorescence in cryosections of the infected ear 7 weeks after *L*. *mexicana* infection. c-MET was expressed in the epidermis as well as in the dermis including expression in neutrophils (**Figs 3B and S4**), Colocalized c-MET with neutrophils is shown in a three-dimensional representation (**Fig 3B**, enlargement). Around 30% of neutrophils were expressing c-MET as quantified by Imaris (**Fig 3C**). Moreover, not only was the protein present, but the c-MET pathway was shown to also be active in these cells, as observed by immunofluorescence staining of phospho-c-MET (P-c-MET) in Ly6G^+^ cells (**Fig 3D**). Colocalized P-c-MET with neutrophils is shown in a three-dimensional representation (**Fig 3D**, enlargement). Similarly, we were able to detect activation of this pathway in MR^+^ dermal macrophages (**Fig 3E**). These data demonstrate the activity of this kinase in cutaneous *Leishmania* lesions.

We then assessed if the level of HGF, the only known ligand for c-MET, was regulated at the site of infection. To this end, C57BL/6 mice were injected i.d. with *L*. *mexicana* metacyclic promastigotes. Three weeks post-infection with 10^4^ metacyclic *L*. *mexicana*, when the first wave of neutrophil is starting at the site of infection with the onset of a lesion (mostly redness) and seven weeks post infection, when the cutaneous lesion is well established, the presence of HGF was analysed by ELISA in ear lysates. A significant increase in HGF levels was observed in *L*. *mexicana* infected ears compared to the levels observed in the ears of non-infected naive mice (**Fig 3F**). In contrast, no significant difference in the levels of serum HGF was observed between uninfected and infected mice (**Fig 3G**) indicating a local, rather than a systemic regulation of c-MET during infection.

### c-MET expression has a minor impact on the effector functions of *L*. *mexicana* infected neutrophils

**Fig 4:**
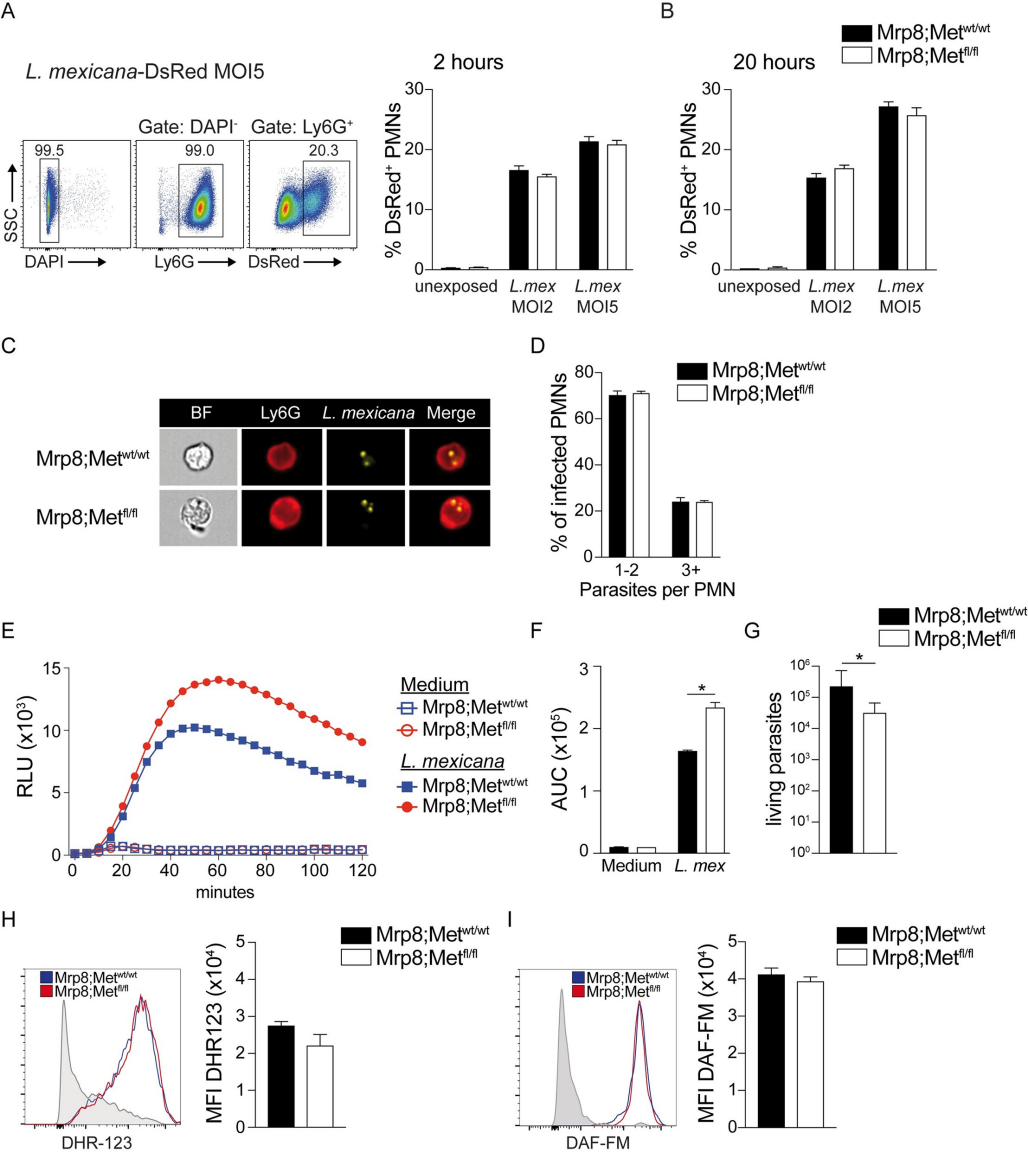
Impact of c-MET on neutrophil functions. BMNs were isolated from mice genetically deficient for c-MET in PMNs (Mrp8;Met^fl/fl^) and control littermate (Mrp8;Met^wt/wt^). PMNs were co-cultured *in vitro* with *L*. *mexicana-*DsRed at the indicated MOI. **A**) 2h post infection free parasites were extensively washed and internalization was assessed by flow cytometry. A representative flow cytometry plot and the frequency of infected PMNs are shown. **B**) A similar analysis was performed 20 hours after infection. **C)** Representative images taken by imaging flow cytometry representing *L*. *mexicana* parasites in Mrp8;Met^fl/fl^ and control Mrp8;Met^wt/wt^ BMNs 24 hours after *L*. *mexicana* infection. **D)** Quantitative assessment of the number of parasites in Mrp8;Met^fl/fl^ and control Mrp8;Met^wt/wt^ neutrophils, 24 hours after infection. **E)** To analyse ROS production, PMNs were exposed for 2h to *L*. *mexicana* parasites at MOI of 5. As control, PMNs similarly grown in medium were analysed. Luminol was added to the co-culture and the chemiluminescence was measured for 2h to evaluate ROS production. Data are shown as relative light units (RLU). **F**) The corresponding area under the curve (AUC) is shown. **G)** Limiting dilution analysis of Mrp8;Met^fl/fl^ and Mrp8;Met^wt/wt^ neutrophils infected with *L*. *mexicana* (MOI 2). **H)** Mrp8;Met^fl/fl^ and Mrp8;Met^wt/wt^ mice were infected with 10^6^ *L*. *mexicana* metacyclic promastigotes in the ear dermis. 24 hours later, the infected ears were collected, and ROS production was assessed by flow cytometry in CD45^+^CD11b^+^Ly6G^+^ PMNs using the DHR123 probe. A representative flow cytometry plot, and the corresponding MFI (right) of a representative experiment are shown. Shaded histograms are the FMO control for DHR123. **I**) NO levels were analyzed by flow cytometry in Mrp8;Met^fl/fl^ and Mrp8;Met^wt/wt^ neutrophils 24 hours after infection. Data are representative of ≥ 2 experiments, n≥3/group.

c-MET expression was previously shown to impact neutrophil function in the tumor environment [7]. To assess if c-MET expression and activation had an impact on *L*. *mexicana*-induced neutrophil effector functions, BMNs derived from mice genetically deficient for c-MET in neutrophils (Mrp8;Met^fl/fl^) and control littermate mice (Mrp8;Met^wt/wt^) were isolated and exposed to metacyclic *L*. *mexicana*-DsRed parasites for 2 and 20 hours at an MOI of 2 and 5. Flow cytometry analysis showed a comparable frequency of infected DsRed^+^ neutrophils between both groups 2 hours post infection (**Fig 4A**) suggesting similar parasite internalisation. The frequency of intracellular parasites present in neutrophils 20h post-*L*. *mexicana*-DsRed infection was analyzed by flow cytometry to determine if the parasite presence was impacted in absence of c-MET. A 16% and 26% frequency of infected neutrophils was observed at MOI2 and MOI5, respectively. However, no difference in the frequency of infected neutrophils was observed between c-MET deficient and control neutrophils (**Fig 4B**). No differences in neutrophil survival and apoptosis were observed between Mrp8;Met^fl/fl^ and Mrp8;Met^wt/wt^ neutrophils at 2 and 20 hours post infection **(S1B Fig).** To assess if the absence of c-MET would have an impact on the parasite number per neutrophils, BMNs from WT (Mrp8;Met^wt/wt^) and c-MET deficient (Mrp8;Met^fl/fl^) neutrophils were infected and 16 hours later analysed by imaging flow cytometry. No difference in parasite number was observed between c-MET deficient and control neutrophils (**Fig 4C and 4D**), confirming our results obtained by conventional flow cytometry.

ROS production is one of the major defence mechanisms of neutrophils, we thus analysed a potential impact of c-MET expression on ROS production. BMNs isolated from the femurs of Mrp8;Met^fl/fl^ or of Mrp8;Met^wt/wt^ mice, were exposed to *L*. *mexicana* metacyclic promastigotes for 2h at MOI of 5. ROS production was measured by the chemiluminescence induced by the reaction of luminol with ROS and analyzed over 120 min. Unexposed neutrophils were used as control. Higher levels of ROS were produced by neutrophils deficient for c-MET compared to WT neutrophils (**Fig 4E and 4F**). As ROS may impact parasite survival, neutrophils were similarly exposed to *L*. *mexicana* and limiting dilution analysis performed. A small but statistically significant decrease in parasite survival was observed in c-MET-deficient neutrophils (**Fig 4G**), suggesting that even though a similar number of DsRed-*L*. *mexicana* is observed in c-MET deficient and WT neutrophils, there appears to be a reduced fitness of parasites present in c-MET deficient neutrophils. These data correlate with higher ROS production observed in c-MET-deficient neutrophils.

We then measured *in vivo* the production of ROS in c-MET deficient and control neutrophils at the site of infection. Mrp8;Met^fl/fl^ and Mrp8;Met^wt/wt^ mice were infected in the ear dermis with 10^6^ *L*. *mexicana* metacyclic promastigotes, and ROS production was assessed in neutrophils 24 hours later by flow cytometry, at a time when neutrophil presence peaks following infection [8]. ROS production in CD45^+^CD11b^+^Ly6G^+^ neutrophils was analyzed using the DHR123 probe. No difference was observed between c-MET deficient and WT neutrophils (**Fig 4H**). The level of nitric oxide (NO) was also assessed by flow cytometry using the DAF-FM probe. No difference between Mrp8;Met^fl/fl^ and Mrp8;Met^wt/wt^ mice was observed (**Fig 4I**). These findings indicate that c-MET expression on neutrophils has no impact on *L*. *mexicana* internalisation and neutrophil effector function except a small effect observed in ROS production *in vitro*, a phenomenon that is compensated *in vivo*.

### Specific deletion of c-MET in neutrophils has a major impact on *L*. *mexicana* lesion development

**Fig 5:**
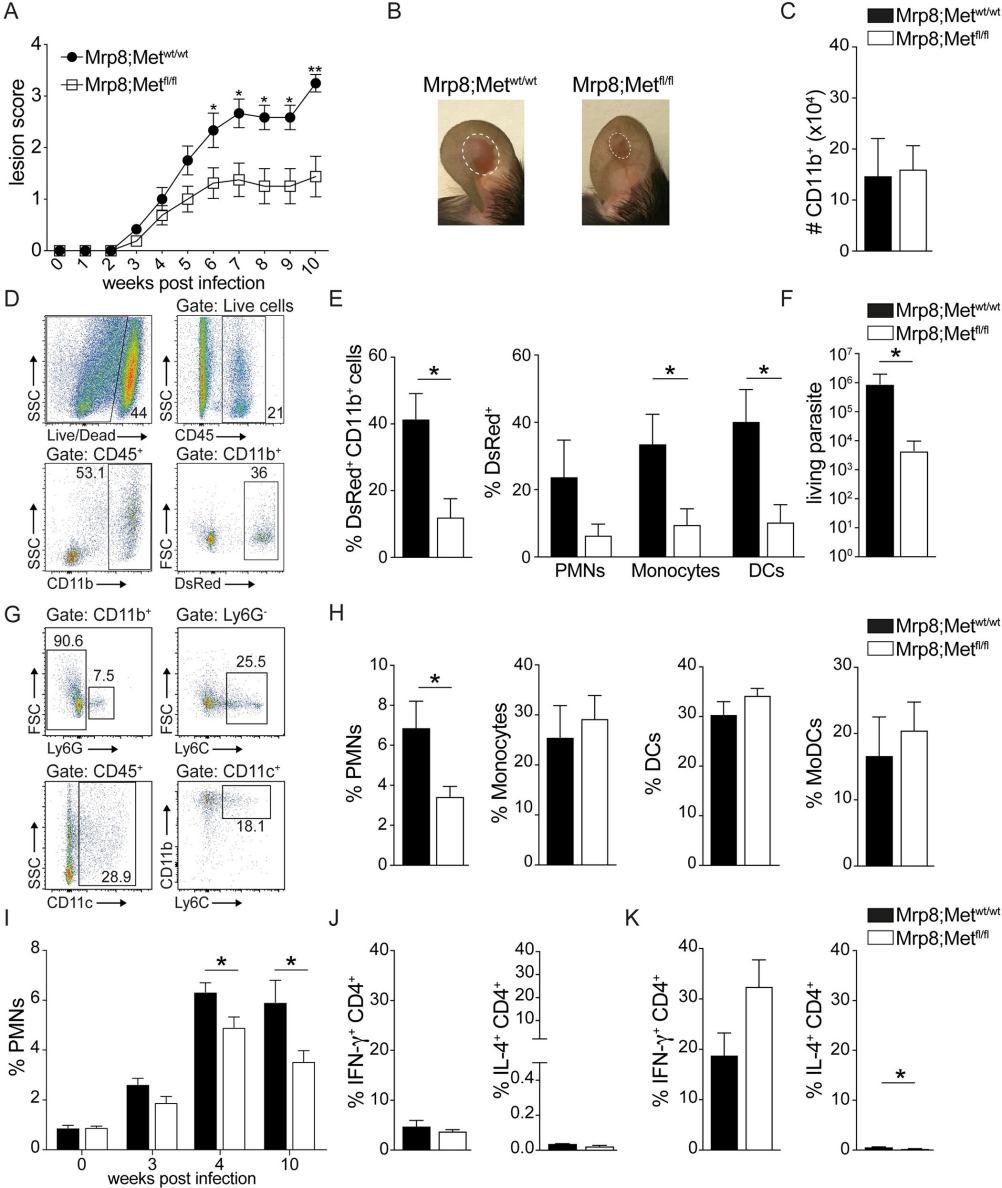
Specific deletion of c-MET in neutrophils has a major impact on *L*. *mexicana* lesion development. Mrp8;Met^fl/fl^ mice selectively deficient for c-MET in PMNs and Mrp8;Met^wt/wt^ control littermate were infected i.d. with 10^4^ *L*. *mexicana-*DsRed metacyclic promastigotes. **A**) Lesion development was measured on a weekly basis using a caliper and lesion score was determined. Briefly, the score is based on the inflammation of the ear, the lesion size (mm) and appearance of necrosis. **B**) Representative pictures of ear lesions ten weeks post infection are shown. Ten weeks post infection, at the end of the experiment, ears and draining lymph nodes (dLNs) were excised and digested. **C**) The number of CD11b^+^ myeloid cells was assessed by flow cytometry at the site of infection. **D**) Representative flow cytometry plot showing the gating strategy for CD45^+^CD11b^+^DsRed^+^ cells. **E**) The frequency of infected myeloid CD45^+^CD11b^+^DsRed^+^ cells and the frequency of infected CD45^+^CD11b^+^Ly6G^+^DsRed^+^ PMNs, CD45^+^CD11b^+^Ly6G^-^Ly6C^+^DsRed^+^ monocytes and CD45^+^CD11c^+^DsRed^+^ DCs in the ear was assessed by flow cytometry. **F**) The parasite load in the infected ears was determined by limiting dilution assay (LDA). **G)** Representative flow cytometry plot of ear derived dermal cells. **H**) Frequency of total CD45^+^CD11b^+^Ly6G^+^ PMNs, CD45^+^CD11b^+^Ly6G^-^Ly6C^+^ monocytes, CD45^+^CD11c^+^ DCs and CD45^+^CD11c^+^Ly6C^+^CD11b^+^ monocyte derived DCs (MoDCs). **I**) The frequency of CD45^+^CD11b^+^Ly6G^+^ PMNs in the ears of Mrp8;Met^fl/fl^ mice and Mrp8;Met^wt/wt^ control littermates was analysed by flow cytometry 0, 3, 4 and 10 weeks post i.d. infection with 10^4^ metacyclic *L*. *mexicana*. The data in **I** are pooled from two independent experiments involving ≥6 mice/ group **J**) The frequency of CD45^+^CD4^+^IFN-γ^+^ and CD45^+^CD4^+^IL-4^+^ T cells in dLNs and **K**) in the ears is presented. Data are representative of ≥3 experiments, n≥6/group, *p <0.05; **p <0.01.

Early neutrophils infiltration in *L*. *mexicana* lesion contributes to the cutaneous pathology [8]. Thus, we hypothesised that the deletion of c-MET in neutrophils might decrease their presence in infected tissues and thereby be beneficial to the disease. To assess the impact of c-MET in neutrophils on the disease outcome, Mrp8;Met^fl/fl^ and Mrp8;Met^wt/wt^ mice were infected i.d. with *L*. *mexicana-*DsRed metacyclic promastigotes. Following inoculation of a high dose (10^6^) of *L*. *mexicana*, elevated HGF levels were observed in ear lysates 24 hours p.i. with similar levels observed between PBS or *L*. *mexicana* injected ears, suggesting that the needle injection itself is inducing HGF release. Accordingly, no difference in neutrophil recruitment was observed at that time point between mice injected either with PBS or a high dose of *L*. *mexicana* (**S5 Fig**). Thus, to analyse the impact of the parasite infection on c-MET activation on neutrophils and its effect on the disease, and to be closer to the physiological parasite dose inoculated during a sand fly blood meal, we infected mice with a low dose (10^4^) of parasites. Parasite-specific neutrophil recruitment to the infected ear takes place between 3–4 weeks p.i. Following infection, Mrp8;Met^fl/fl^ and Mrp8;Met^wt/wt^ control mice developed a small lesion that did not differ until 3–4 weeks post infection. From there on, Mrp8;Met^fl/fl^ mice controlled better the infection and developed a significantly smaller lesion than similarly infected Mrp8;Met^wt/wt^ control mice (**Fig 5A and 5B**). Ten weeks post-infection, the number of CD11b^+^ cells recruited in the infected ear was comparable between the two groups of mice (**Fig 5C**) but the frequency of infected (DsRed^+^) cells in the CD11b^+^ population was significantly reduced in Mrp8;Met^fl/fl^ infected ears (**Fig 5D and 5E**). Accordingly, neutrophils, monocytes and dendritic cells were less infected in Mrp8;Met^fl/fl^ compared to control Mrp8;Met^wt/wt^ mice (**Fig 5E**). In addition, the parasite load in the infected ear, as determined by limiting dilution analysis (LDA), was significantly lower in Mrp8;Met^fl/fl^ than in Mrp8;Met^wt/wt^ infected ears (**Fig 5F**).

The ear cellularity at the infected site was analysed by flow cytometry (**Fig 5G**). The frequency of neutrophils recruited to the ear dermis was significantly lower in Mrp8;Met^fl/fl^ than Mrp8;Met^wt/wt^ mice at ten weeks post-infection, whereas no differences were observed in the frequency of monocytes, DCs and monocytes derived DCs (MoDC) (**Fig 5H**). To further demonstrate the importance of c-MET in neutrophil recruitment to the site of infection, we also analyzed the frequency of ear-recruited neutrophils early after infection, when no difference in lesion size was yet observed between Mrp8;Met^fl/fl^ and Mrp8;Met^wt/wt^ mice. Ears were isolated, digested and neutrophil presence was analyzed by flow cytometry. Only a very low neutrophil frequency was observed in Mrp8;Met^fl/fl^ and Mrp8;Met^wt/wt^ ears at basal levels (time 0) which was similar between both groups. Three weeks post infection, a few neutrophils started to be recruited to the site of infection, and already a tendency to a lower frequency of recruited neutrophils was observed in Mrp8;Met^fl/fl^ compared to Mrp8;Met^wt/wt^ ears. Four weeks post infection, higher levels of neutrophils were recruited to the infected site and a significant decrease in neutrophil recruitment was observed in Mrp8;Met^fl/fl^ compared to Mrp8;Met^wt/wt^ ears. Ten weeks post infection, the difference in neutrophil frequency observed between Mrp8;Met^fl/fl^ and Mrp8;Met^wt/wt^ infected ears was further increased (**Fig 5I**).

To analyse the type of adaptive immune response developing in these mice, we measured the frequency of CD4^+^IFN-γ^+^ and CD4^+^IL-4^+^ cells present in the draining lymph node (dLN) and infected ear. In the dLN, the frequency of CD4^+^ T cells expressing IFN-γ and IL-4 was similar between the two mouse strains (**Fig 5J**). At the site of infection, an increase in the frequency of CD4^+^IFN-γ^+^ T cells was consistently observed in the ears of Mrp8;Met^fl/fl^ compared to control mice, although it was not statistically significant in all experiments (**Fig 5K**). Correspondingly, the frequency of CD4^+^IL-4^+^ T cells was lower in the ears of Mrp8;Met^fl/fl^ compared to control group (**Fig 5K**). Collectively, our results indicate that c-MET signalling in neutrophils is contributing to the development of the pathology associated with *L*. *mexicana* infection.

### Pharmacological systemic c-MET inhibition, once the *L*. *mexicana* lesion is established, leads to a decrease in lesion size and neutrophil infiltration

**Fig 6:**
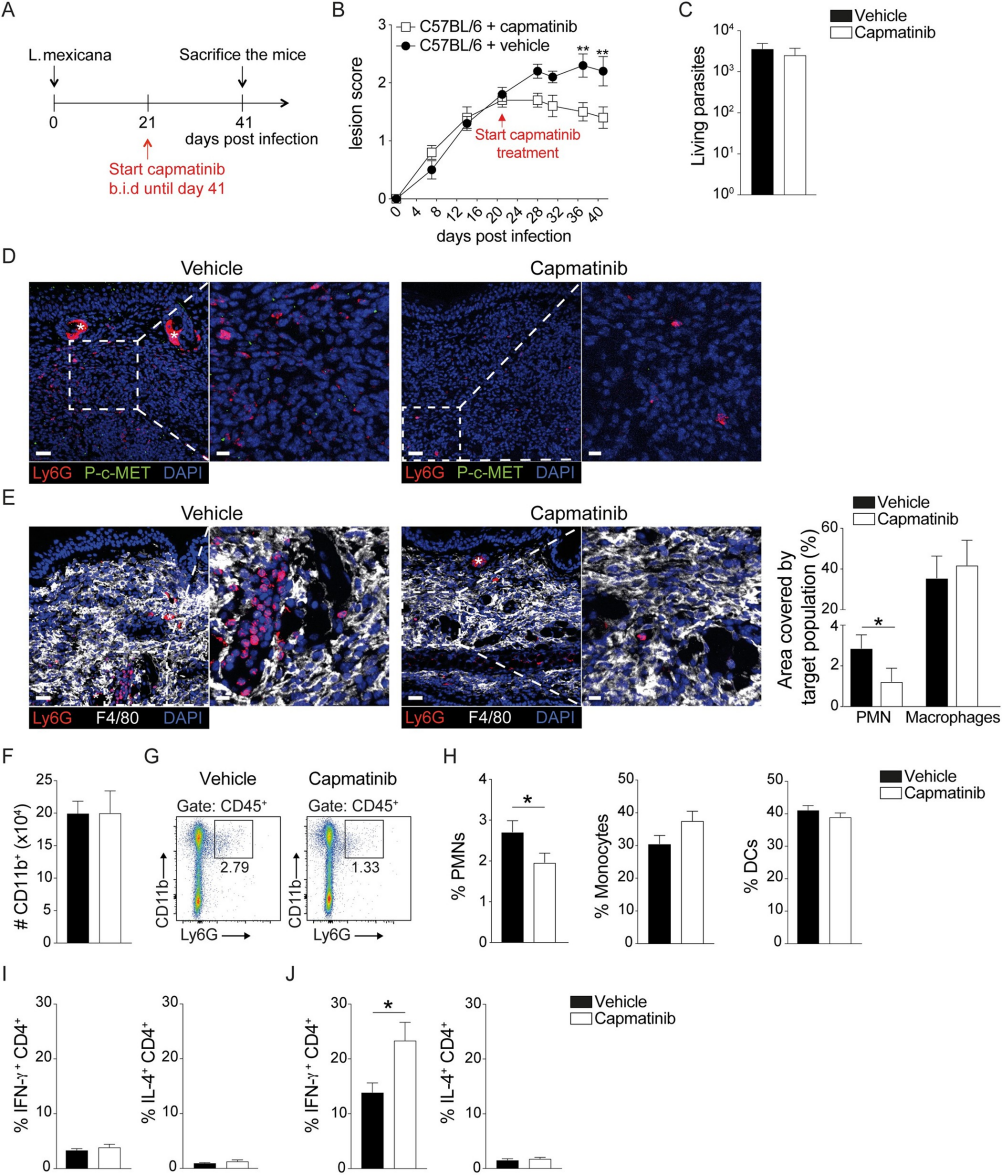
Systemic c-MET inhibition leads to decreased lesion size and inflammation during *L*. *mexicana* infection. C57BL/6 mice were infected with 10^6^ *L*. *mexicana* metacyclic promastigotes in the ear dermis. After twenty-one days, mice received 5mg/kg capmatinib intraperitoneal (i.p.) twice a day (*bis in die (b*.*i*.*d*.)) for twenty consecutive days. Control mice were injected with vehicle. **A**) Experimental strategy. **B**) The lesion score of mice treated or not with capmatinib is shown over 40 days. **C**) The ears of infected mice were collected and processed after twenty days of capmatinib or vehicle treatment. Parasite burden in the ear was analysed by limiting dilution assay (LDA). **D**) Representative immunofluorescence staining and confocal microscopy of a representative section of an infected ear twenty days post treatment, showing staining for Ly6G^+^ neutrophils (red), phosphorylated (P) c-MET (green) and DAPI (blue). Scale bars: 30 μm, stars show hair follicles. **E**) Immunofluorescence staining for Ly6G^+^ (red) neutrophils, F4/80^+^ macrophages (white) and DAPI^+^ nuclei (blue) in infected ears treated or not with capmatinib; scale bars: 30 μm, stars show endogenous biotin (left). The corresponding quantification of the area covered by Ly6G^+^ neutrophils and F4/80^+^ macrophages in capmatinib and control-stained ears (right) as assessed by IMARIS is shown. **F**) The number of myeloid CD11b^+^ cells in the infected ears was assessed by flow cytometry. **G**) Representative gating strategy for ear-derived neutrophils. **H**) The frequency of CD45^+^CD11b^+^Ly6G^+^ PMN, CD45^+^CD11b^+^Ly6G^-^Ly6C^+^ monocytes and CD45^+^CD11c^+^ DCs at the site of infection is given. **I**) The frequency of CD45^+^CD4^+^ IFN-γ^+^ and CD45^+^CD4^+^ IL-4^+^ T cells in the dLNs and **J**) ears is shown. Data are representative of ≥2 experiments, n≥4/group, *p <0.05; **p <0.01.

Patients seek treatment when they harbour a defined cutaneous lesion. To translate our results to potential clinically relevant settings, c-MET signalling was systemically blocked once a cutaneous lesion was established in mice. To this end, C57BL/6 mice were infected i.d. with *L*. *mexicana* metacyclic promastigotes and twenty-one days post infection mice received the c-MET inhibitor capmatinib intraperitoneally twice a day (*b*.*i*.*d*.) for 20 consecutive days. Control mice received the vehicle solvent used to dilute the capmatinib as illustrated (**Fig 6A**). Already 4 days post treatment, we observed a decrease in lesion size in mice receiving capmatinib injection compared to control mice, that became statistically significant 15 days post initiation of the treatment and remains so until the end of the treatment. (**Fig 6B**). The parasite load present in the infected ears at the end of the treatment, as analysed by limiting dilution assay (LDA), showed comparable parasite burden between the two groups (**Fig 6C**). Immunofluorescence of ear cryosection confirmed the success of the treatment, as the phospho c-MET signal observed in the vehicle group was almost abolished in the capmatinib treated group (**Fig 6D**). Furthermore, a significant decrease in the Ly6G^+^ area at the site of infection was observed in mice treated with the c-MET inhibitor, correlating with the results obtained with mice genetically deficient in c-MET in their neutrophils. Interestingly, the area covered by macrophages was similar between the two groups of mice, indicating that the systemic treatment affected specifically the presence of neutrophils at the lesion site (**Fig 6D and 6E**). To further quantify the infiltration of immune cells upon c-MET inhibition, we assessed the frequency of recruited cells at the site of infection by flow cytometry. We found an equal number of CD11b^+^ cells in mice treated or not with the c-MET inhibitor (**Fig 6F**). In line with the immunohistology data, we detected a significant decrease in neutrophil infiltration in the ears of capmatinib-treated mice (**Fig 6G and 6H**). In contrast, the frequency of monocytes and dendritic cells was similar in the two groups (**Fig 6H**).

We then analysed if the adaptive immune response was modified at the end of treatment in capmatinib-treated mice. To this end, the frequency of CD4^+^ T cells expressing IFN-γ and IL-4 at the site of infection and in dLN was analysed by flow cytometry. No difference in the frequency of IFN-γ^+^ and IL-4^+^ CD4^+^ T cells was observed in the two groups of mice in the dLN (**Fig 6I**). However, at the site of infection a significant increase in the levels of intracellular IFN-γ was detected in CD4^+^ T cells of capmatinib-treated mice compared to control mice (**Fig 6J**). No difference in the low frequency of CD4^+^IL-4^+^ T cells was observed in the infected ears. Overall, these results indicate that blocking c-MET once a lesion is established, impairs further recruitment of neutrophils, leading to a decrease in lesion size and modifications in local IFN-γ levels without impacting the parasite burden.

## Discussion

c-MET expression in neutrophils has been shown to be critical for their migration during inflammatory settings. Furthermore, c-MET expression on neutrophils contributes to the killing of tumour cells in the tumour microenvironment [7,19,20]. The level of c-MET in bone marrow leukocytes is rapidly upregulated during inflammation and c-MET was reported to regulate the egress of cells from the bone marrow [21]. In the same line, the low levels of c-MET in circulating human and mouse neutrophils were shown to be upregulated following inflammatory stimuli [7]. The classical mechanism of c-MET activation is by its binding to its HGF ligand. In the skin, the major source of HGF comes from fibroblasts [22], however, neutrophils can also produce HGF [23–25], which is pre-stored in secretory vesicles and gelatinase granules [25]. In response to *L*. *mexicana* infection *in vitro*, neutrophils upregulated c-MET despite the lack of detectable HGF levels in the culture supernatant. Furthermore, c-MET expression was significantly more induced in infected *versus* non-infected bystander neutrophils. In contrast, infection of DCs and macrophages did not induce significantly c-MET expression *in vitro*. These results demonstrate that the parasite itself induces c-MET expression in neutrophils. Interestingly, this induction was also observed in other *Leishmania* spp such as *L*. *major*, suggesting that *Leishmania-*induced c-Met expression contributes to neutrophil recruitment in leishmaniasis. Other pathogens including bacteria, virus and other parasites were also shown to promote c-MET activation in target cells [26–30]. In addition, c-MET activation was required for the infection and survival of *Plasmodium* sporozoites in hepatocytes [27,31]. In bacteria, the virulence factor internalin B (InlB) of *Listeria monocytogenes* was reported to bind to the extracellular region of c-MET expressed on epithelial cells, promoting bacterial internalization [26,32]. Similarly, *Salmonella* (*S*.) *typhi* express the virulence factor *S*. *typhi* invasin (STIV), which directly binds c-MET, favouring epithelial cell invasion [30]. Here, using flow cytometry imaging and flow cytometry, we show that c-MET expression in neutrophils has no impact on their parasite internalisation. In chronic infected inflammatory lesions, cutaneous damage at the site of infection induces HGF release. In line with this, we observed c-MET tyrosine phosphorylation at the site of infection in neutrophils as well as in dermal macrophages.

It has been previously shown in cancer that c-MET expression in neutrophils is regulated by TNFα-dependent NF-κB activation [7]. c-MET expression could be induced by intracellular signalling pathways activated directly by the parasites. In this line, the LPS present on Gram-negative bacteria was previously shown to promote c-MET expression in neutrophils through activation of Toll-like receptor 4 (TLR4) signalling [7]. *Leishmania* are recognized by several TLRs such as TLR2, 4, 7 and 9 [33–41], suggesting that c-MET upregulation in lesional neutrophils may be driven by direct triggering of TLR signalling pathway by *L*. *mexicana*. However, induction of c-MET was not linked to TLR4 or other TLR triggering by *L*. *mexicana*, as MyD88^-/-^, TLR4^-/-^ and WT BMNs exposed to *L*. *mexicana* were equally capable to induce c-MET surface expression. We further show that activation of the NF-κΒ pathway contributes to the induction of c-MET on neutrophils by *L*. *mexicana*. These data are in line with a previous report showing that LPS- and TNF- mediated c-MET induction required NF-κΒ activation[7].

Cell traversal (hepatocytes) observed in several species of *Plasmodium*, the causative agent of malaria, was reported to induce HGF secretion, the activation of c-MET by hepatocytes and invasion of these cells [27], while other *Plasmodium* spp did not [42]. *L*. *mexicana* induced the expression of c-MET on neutrophils but did not show any impact on the phagocytosis of the parasites. The production of radical oxygen species (ROS) by neutrophils is one of the major effector mechanisms of neutrophil killing. Infection of Mrp8;Met^fl/fl^ c-MET-deficient neutrophils resulted in increased ROS release compared to their wild type counterpart. This led to a small but statistically significant decrease in live parasites released by *in vitro* infected neutrophils. These data suggest that c-MET activation on neutrophils decreases ROS production by infected neutrophils. However, *in vivo*, following infection with a high dose of parasites, the impact of c-MET expression on ROS production by neutrophils is compensated by other local activating factors of neutrophils. Similarly, no difference in NO production was observed 24 hours after infection.

Numerous factors, including the dose of parasites injected and the inflammation caused by the local tissue damage are involved in the first wave of neutrophil recruitment following i.d. *Leishmania* inoculation [8,40,43,44]. Of note, any tissue damage caused by sand fly bite or needle injection will induce HGF/c-MET signalling to promote the wound healing of the skin. To investigate the function of *L*. *mexicana*-induced c-MET activity selectively in neutrophils, we infected mice with a low dose of parasites, a process inducing a parasite-dependent neutrophil recruitment around three weeks post infection, at a time when the multiple neutrophil-attracting factors induced by the physical damage to the ear do not persist. We showed that the onset of parasite-induced neutrophil recruitment observed from three weeks post infection correlated with increased HGF levels at the site of infection. In addition, Mrp8;Met^fl/fl^ mice infected with a low dose of parasites controlled better their lesion development and harboured decreased parasite burden compared to control mice similarly infected. Importantly, impaired neutrophil recruitment was observed already early in infection, at a time when neutrophils start to infiltrate the site of infection in response to parasites, but when the ear inflammation is barely detectable and no difference in lesion score is apparent between Mrp8;Met^fl/fl^ and Mrp8;Met^wt/wt^ ears. These data demonstrate that the absence of c-MET in neutrophils contributes to the reduction in neutrophil recruitment observed throughout the infection.

An increase in the frequency of CD4^+^IFNγ^+^ T cells was observed at the site of infection both in mice genetically deficient for c-MET in neutrophils and in the lesion of mice treated with the c-MET inhibitor capmatinib. These results suggest a suppressive role of c-MET in the effector function of CD4^+^IFNγ^+^ T cells at the site of infection. An effect of c-MET expression on neutrophils on the T cell effector functions was previously reported for CD8^+^ T cells in an experimental model of cancer, where the fitness of adoptively transferred CD8^+^ T cells in Mrp8;Met^fl/fl^ mice was enhanced [19]. The increase in CD4^+^IFNγ^+^ T cells and the decrease in CD4^+^IL-4^+^ T cells observed at the site of infection correlated with decreased parasite burden in the lesion of Mrp8;Met^fl/fl^ mice. Collectively, our data suggest that c-MET expression on neutrophils also contributes to the impairment of the local adaptive immune response.

Despite increased levels of IFNγ, decreased parasite burden was not observed in mice following systemic inhibition of c-MET signalling once a cutaneous lesion is established, this could be linked to the length of the treatment. A major difference between Mrp8;Met^fl/fl^ mice and mice treated with the c-MET inhibitor capmatinib, is that pharmacological inhibition of c-MET acts on all cells, while in Mrp8;Met^fl/fl^ mice, impaired c-MET expression is restricted to neutrophils. We observed induced expression of phospho-c-MET in dermal macrophages at the lesion site, showing c-MET activation in dermal macrophages. Inhibition of c-MET in hematopoietic cells was previously shown to decrease total NO levels at the tumour site, a process correlating with decreased iNOS by neutrophils [7]. We did not observe *L*. *mexicana*-induced NO production in neutrophils but the global pharmacological inhibition of c-MET *in vivo* could impair either directly or indirectly the IFNγ-induced NO production by macrophages, a process critical in killing the parasites.

In summary, our results reveal that c-MET expression is activated in neutrophils during *L*. *mexicana* infection. This favours their migration to the site of infection, providing a protective niche for the parasites that can further expand in the host, contributing to the chronicity of the disease. Furthermore, we showed that c-MET activation on neutrophils exacerbates the cutaneous pathology, prompting us to treat the cutaneous lesion with a c-Met inhibitor, with significant impact on the pathology. Collectively, our data reveal a potential use for c-MET inhibitors in the control of the cutaneous *Leishmania* lesion.

## Material and methods

### Ethics statements

Animal experimentation protocols were approved by the veterinary office of the Canton of Vaud (Authorization 1266.6–7 to F.T.C.) and were done in accordance to cantonal and federal law as well as the principles of the declaration of Basel.

### Mice

C57BL/6 mice were purchased from Envigo (Cambridgeshire, United Kingdom). Mrp8;Met^fl/fl^ (Cre-positive) and Mrp8;Met^wt/wt^ (Cre-negative) on C57BL/6 background were provided by Professor Massimiliano Mazzone (University of Leuven) [7]. MyD88^-/-^ and TLR4^-/-^ mice on C57BL/6 background were obtained from Prof. Shizuo Akira (University of Osaka). Mice were bred in pathogen-free conditions at the Epalinges centre. 6–8 weeks-old female mice were used in the experiments.

### Parasites

*L*. *mexicana* (MYNC/BZ/62/M379) WT, *L*. *mexicana*-DsRed [45] and *L*. *major* LV39 (MRHO/SU/59/P) [40] were used. Parasites were cultured at 26°C in M199 medium (Gibco) supplemented with 10% fetal calf serum (FCS), 4% Hepes and 2% penicillin-streptomycin-neomycin (PSN). Transgenic DsRed parasites were growth in the same media supplemented with 50 μg/mL of Hygromycin B (Sigma-Aldrich). Metacyclic promastigotes were isolated from stationary phase parasites as previously described [8]. Amastigotes were isolated from footpad lesions of mice infected for >8 weeks as previously described [17]. Briefly, infected footpads were mechanically homogenized in MEG buffer, debris were eliminated by centrifugation and amastigotes were filtered through a 40 μm, 8 μm and 5 μm filters (BD Bioscience). Axenic amastigotes were obtained after 3 days of incubation of stationary-parasite parasites, at 34°C in complete M199 media supplemented with 10% sodium phosphate buffer (100mM, pH 5.5). For *in vivo* infection metacyclic promastigotes were needle injected in the ear dermis, lesion development was measured weekly using a caliper as previously described [46]. A score between 0 and 8 is attributed according to inflammation, lesion size and ear integrity. Briefly, naïve ears have a score of 0, whereas the appearance of inflammation indicates a score of 0.5. When the lesion is delimited the caliper is used to measure the lesion size, the appearance of necrosis is scored as 6, whereas 8 defines the tissue destruction.

Dead *L*. *mexicana* parasites were obtained after submitting promastigotes to seven freeze-thaw cycles.

### c-MET inhibition

c-MET inhibitor capmatinib (INC280 Selleck Chemicals) was used to block c-MET *in vivo*. Mice were given 5 mg/kg of capmatinib intraperitoneal (i.p.) twice a day (*b*.*i*.*d*) during the indicated time as previously described [19]. Control mice were injected with the vehicle solvent used to dilute the capmatinib.

### Immunofluorescence

Ears were collected, fixed in 4% PFA at 4°C overnight and washed. Then, they were cryoprotected in 30% and embedded in Tissue-Tek OCT (Sakura). 20- or 8-μm thick OCT sections were thawed for 30 minutes at room temperature, fixed in 4% PFA for 10 minutes and permeabilized with 0.3% Triton X-100 in PBS. After 30 minutes in blocking buffer (0.5% BSA, 5% donkey serum, 0.3% Triton X-100, 0,1% NaN3), slides were incubated with primary antibodies overnight at 4°C. The next day slides were washed with 0.3% Triton X-100 in PBS for 30 minutes and appropriate alexa secondary antibodies 488/555 were added 1:500 in blocking buffer for 1 hour at RT. After that, slides were washed with 0.3% Triton X-100 in PBS for 30 minutes and mounted.

The following antibodies were used: either unlabelled or biotin-labelled Ly6G (Biolegend), anti-F4/80-APC (Invitrogen), anti-MET (Abcam), anti-Phospho-MET (Tyr1234/1235) (Cell Signaling Technology), anti-CD206 (Biolegend). Images were acquired on a Zeiss LSM880 and analysed using Imaris. Quantification of Ly6G^+^ and F4/80^+^ cells was performed using ImageJ software. 3D rendering and visualisation were also generated using the Imaris cell imaging software.

### Bone marrow-derived myeloid cells

Myeloid cells were isolated from the bone marrow (BM) of naïve female mice. Femora and tibia were collected and BM was obtained by flushing the bones with RPMI media. Neutrophils were purified by positive magnetic-activated cells sorting (MACS) using anti-Ly6G MicroBeads UltraPure isolation kit, according to manufacturer’s instructions (Miltenyi Biotec). The purity of neutrophils (>95%) was assessed by cytospin assay or flow cytometry. BM progenitor cells were differentiated for 6 days in 20% L929-MCSF supernatant to generate BM-derived macrophages. BM progenitor cells were differentiated for 7 days in 20% AO2-GMCSF, in order to obtain BM-derived dendritic cells.

When indicated, cells were treated with 50 ng/mL of LPS from *E*. *coli* O127:B8 (Sigma-Aldrich). For NF-κΒ inhibition, neutrophils were pre-treated for 1h at 37°C with 10μM of IKK inhibitor III (BMS-345541) (Selleck, EU).

### mRNA isolation and real-time PCR

Myeloid cells were lysed in RLT buffer, and mRNA was extracted using the RNeasy Plus Mini kit (Qiagen) according to manufacturer’s instructions. 500 ng of mRNA was reverse transcribed into cDNA using random nonamers primers (Microsynth) and M-MLV RT polymerase (Promega). cDNA was then purified using the QIAquick PCR purification kit (Qiagen) according to manufacturer’s instructions. c-MET gene expression was assessed by Real-time PCR using the SYBR green kit (Roche) and the LightCycler system (Roche). c-MET expression was normalized to the housekeeping gene hypoxanthine phosphoribosyl transferase (HPRT), using the comparative threshold cycle method for relative quantification. The following primers (Microsynth) were used: c-MET Forward: 5’-CTGCTCTGGAGGACAAGACC-3’, Backward: 5’- GAGTTGATCACATGCCAAGC-3’; HPRT Forward: 5’- GTTGGATATGCCCTTGAC-3’, Backward 5’-AGGACTAGAACACCTGCT-3’.

### Isolation of ear and lymph node mouse cells

Ears were collected at indicated time points and processed to obtain a single cell suspension as previously described [41]. Briefly, the two dermal layers were separated, homogenized and digested at 37°C for 2h in 0.2 mg/mL of liberase TL (Roche). The digested ears were filtered using 40μm filters (Falcon) and a cell suspension obtained. Draining lymph node (dLN) were recovered and homogenized using a glass homogenizer to obtain a single cell suspension.

### Flow cytometry

Stained cells were analyzed using LSR-Fortessa (BD Bioscience) and analysed with FlowJo software (Tree Star). The following antibodies were used to detect cell surface expression: anti-CD45-PerCPCy5.5, anti-CD11c-PeCy7, anti-Ly6C-FITC, anti-Ly6G-PE and anti-Ly6C-APC (BD Bioscience); anti-Ly6G-APC/Cy7 and anti-CD4-AF700 (Biolegend); anti-CD8-APC, anti-CD11b-PB, anti-IFNγ-PeCy7 and c-MET-FITC (Invitrogen); anti-IFNγ-PE, anti-IL-4-FITC (eBioscience) and anti-Annexin V-PeCy7 (eBioscience).

Cell viability was analyzed using the Live/Dead fixable Aqua Dead Cell Stain Kit (Invitrogen) for *ex vivo* experiments, whereas DAPI (Sigma-Aldrich) was used for *in vitro* experiments. Parasite-infected cells were identifies based on the DsRed fluorescence of transgenic parasites.

### Imaging flow cytometry

Samples were analysed using an Image Stream cytometer (Amnis; Millipore Sigma, Billerica) at low speed and 60x magnification. Debris and free parasites were excluded from the acquisition based on the area and aspect ratio of the bright field. Internalized parasites were differentiated from non-internalized parasites according to the internalization of the bright parasite spots within the membrane marker mask. The number of parasites per neutrophil was determined using the spot count within the parasite channel feature. The IDEA software was used for the analysis. Neutrophils were stained with Ly6G-APC/Cy7 (Biolegend) antibody and cell viability was assessed using DAPI (Sigma-Aldrich).

### ROS and NO detection

To measure ROS formation by luminol based chemiluminescence assay, bone marrow neutrophils were incubated in X-vivo (Lonza) medium in a white opaque 96-well plate (White Opaque 96-well Microplate, PerkinElmer) and exposed to *L*. *mexicana*. 20 μg/ml of luminol (Carbosynth) was added to the cells and ROS chemiluminescence was measured over time at all wavelengths using a plate reader (molecular devices, SpectraMax). To measure ROS and NO by flow cytometry, ear cells were incubated with 1.2 μM DHR123 molecular probe (Thermo Fisher Scientific) or 1 μM DAF-FM probe (Invitrogen), respectively for 45 min at 37°C.

### Cytokine detection

Intracellular cytokines were detected by flow cytometry in ears and dLNs. 10^6^ dLN cells or 10^5^ ear cells were stimulated for 4h at 37°C with 50 μg/mL PMA, 500 μg/mL Ionomycin and 1 μg/mL GolgiPlug (BD Bioscience). Cells were stained for extracellular markers and Live/Dead dye, and fixed with 2% PFA. Saponin was used to permeabilize the cells. Intracellular cytokines were stained for 45 min by directly diluting the antibodies in saponin.

### HGF analysis in the ear

Ears were collected, frozen in liquid nitrogen and homogenized in RIPA lysis buffer (Sigma-Aldrich) supplemented with protease inhibitor cocktail (Roche) using a magnetic bead and a tissue lyser (Qiagen). The ear lysate was analysed for HGF by ELISA (R&D systems) according to manufacturer’s instructions.

### Limiting dilution assay

In order to estimate the number of living parasites, single cell suspensions from infected mice were serially diluted in supplemented M199 media. Each dilution was loaded in 8-fold replicates, in a 96-well plate containing 100μl of rabbit blood agar. The plates were cultured at 26°C during 7 days. The number of wells positive for parasites was determined by microscopy, whereas the number of parasites was determined using the ESTIMFRE software as previously described [47].

### Statistical analysis

*P* values were determined using GraphPad Prism version 8. Statistical difference between two groups was analysed using Mann-Whitney nonparametric t-test. Whereas difference between multiple groups were determined using one way analysis of variance (ANOVA).

## Supporting information

S1 Fig Neutrophil survival is not affected by *L*. *mexicana* infection. **A**) BM-derived neutrophils (BMNs) were isolated and exposed for 16h to *L*. *mexicana* metacyclic promastigotes at the indicated multiplicity of infection (MOI). The apoptotic status of control and *L*. *mexicana*-exposed neutrophils was analyzed by flow cytometry using Annexin V and DAPI staining. The frequency of live (AnV^-^DAPI^-^), early-apoptotic (AnV^+^DAPI^-^) and late apoptotic (AnV^+^DAPI^+^) neutrophils is shown. **B**) BMNs were isolated from Mrp8:Met^fl/fl^ and control Mrp8;Met^wt/wt^ littermates and exposed to *L*. *mexicana-*DsRed for 2 or 20h at indicated MOI. The frequency of live (DAPI^-^) BMNs was assessed by flow cytometry. Data are representative of 3 independent experiments with n≥ 2.
(TIF)

S2 Fig *L*. *major* LV39 upregulate c-MET mRNA expression selectively in neutrophils. **A**) BM neutrophils (BMNs) were isolated and co-cultured *in vitro* with metacyclic *L*. *major* LV39 for 16h at a MOI of 5. c-MET mRNA levels were analysed by RT-qPCR. Unexposed BMNs were used as negative control and LPS treated cells as positive control. **B**) BM-derived macrophages (BMMs) and **C**) BM-derived dendritic cells (BMDCs) were similarly infected and c-MET expression was assessed by RT-qPCR. Data are normalized to endogenous levels of HPRT mRNA and expressed as fold increase relative to expression levels measured in control cells. Data are representative of ≥ 3 experiments, n = 3/group.
(TIF)

S3 Fig *L*. *mexicana*-induced c-MET expression in neutrophils is independent of MyD88 and TLR4 signaling and partially induced by NF-κB activation. **A**) BMNs were isolated from MyD88^-/-^ and C57BL/6 mice and co-cultured for 16h with *L*. *mexicana* metacyclic promastigotes at a MOI of 5. c-MET expression in infected or non-infected (unexposed) neutrophils was assessed by flow cytometry. The MFI of a representative experiment is shown. **B)** BMNs isolated from TLR4^-/-^ and C57BL/6 mice were processed as indicated in **A**. The MFI of a representative experiment is shown. **C**) BMN were isolated from C57BL/6 mice and pre-treated for 1h with the IKK inhibitor III. Cells were then exposed to *L*. *mexicana* for 16h. c-MET expression was assessed by flow cytometry. The representative MFI is shown. Data are representative of ≥ 2 experiments, n≥3.
(TIF)

S4 Fig Immunofluorescent controls showing the specificity of c-MET and P-c-MET antibodies and c-MET expression in MR^+^ dermal macrophages. **A**) Representative histology pictures of naïve liver stained with c-MET (green), F4/80 (white) and DAPI (blue) on the left. Liver stained in absence of the first step c-MET and F4/80 antibodies on the right, as control for primary antibodies specificity. Scale bar: 30μm. **B**) Representative pictures of Mrp8;Met^wt/wt^ and Mrp8;Met^fl/fl^ ears 6 hours p.i with 10^6^ *L*. *mexicana*, staining for phospho-c-MET (P-c-MET, green), Ly6G (red) and DAPI (blue). Scale bars: 30μm. Note the absence of P-c-MET staining in neutrophils in Mrp8;Met^fl/fl^ ears. Scale bar: 10μm. **C**) Representative histology picture of six weeks infected ear, showing immunofluorescent staining for MR^+^ dermal macrophages (red), c-MET (green) and DAPI^+^ nuclei (blue). Scale bar: 30μm (left), enlargement of the defined area (right), scale bars: 10μm. Data are representative of ≥1 experiments.
(TIF)

S5 Fig HGF levels and PMNs infiltration in the ear of mice 24h after infection with 10^6^ *L*. *mexicana*. **A**) C57BL/6 mice were infected i.d. with 10^6^ *L*. *mexicana* metacyclic promastigotes. Twenty-four hours late, the infected ears were collected and homogenized in RIPA buffer using a tissue lyser and the level of HGF analysed by ELISA. PBS-injected ears were used as a control. **B**) Mrp8;Met^fl/fl^ mice deficient for c-MET in PMNs and Mrp8;Met^wt/wt^ control littermate were infected with 10^6^ *L*. *mexicana* metacyclic promastigotes in the ear dermis. Twenty-four hours post infection, ears were collected and digested to obtain a cell suspension. The frequency of CD45^+^CD11b^+^Ly6G^+^ PMNs was assessed by flow cytometry. Data are representative of ≥2 independent experiments, n≥5/group.
(TIF)
