# Supplementary figures and images for: The c-MET receptor tyrosine kinase contributes to neutrophil-driven pathology in cutaneous leishmaniasis
Source: PLoS Pathog. 2022 Jan 18;18(1):e1010247. doi: 10.1371/journal.ppat.1010247 (PMC8797216; doi:10.1371/journal.ppat.1010247)

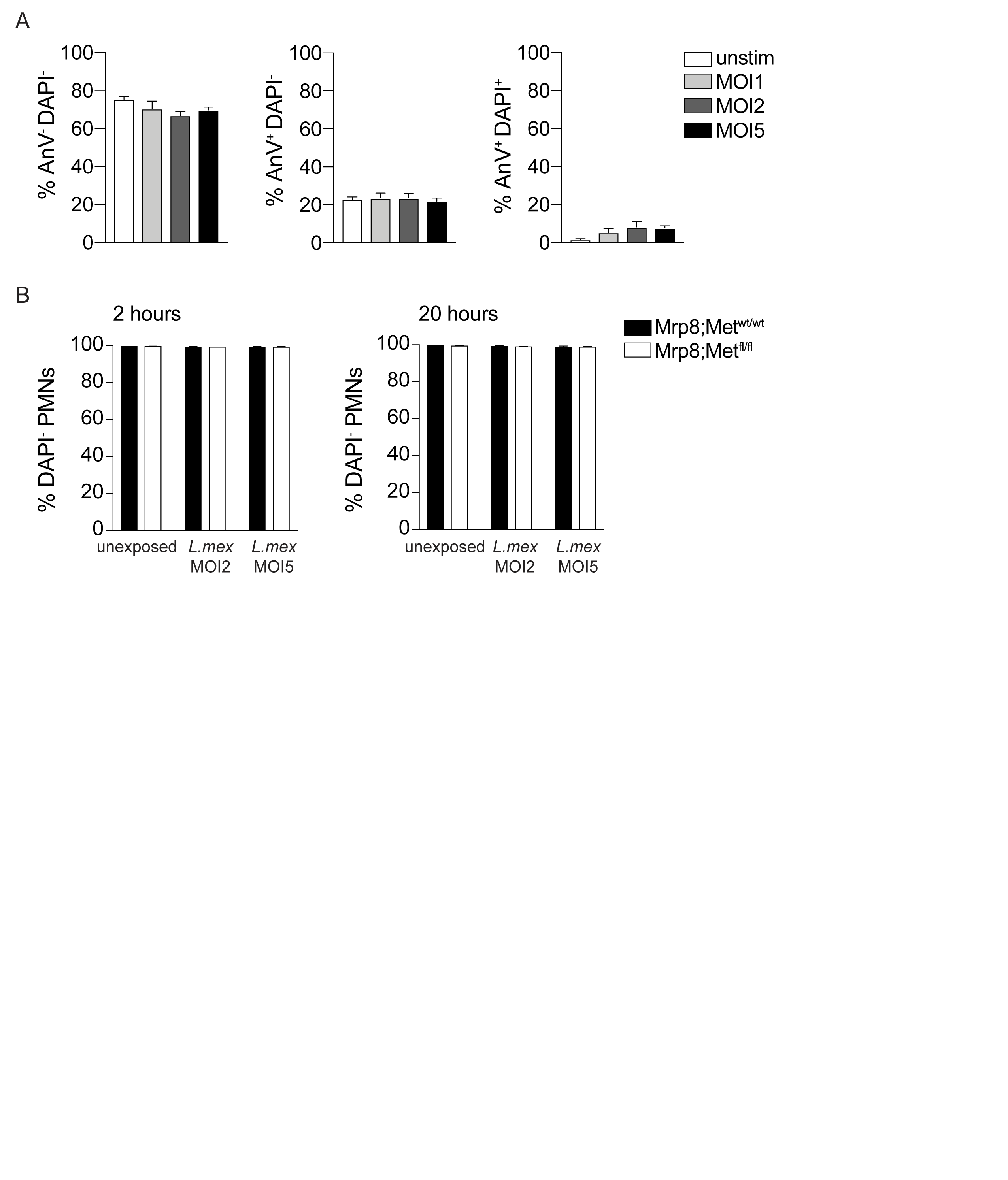

Supplement: S1 Fig — A) BM-derived neutrophils (BMNs) were isolated and exposed for 16h to L. mexicana metacyclic promastigotes at the indicated multiplicity of infection (MOI). The apoptotic status of control and L. mexicana-exposed neutrophils was analyzed by flow cytometry using Annexin V and DAPI staining. The frequency of live (AnV-DAPI-), early-apoptotic (AnV+DAPI-) and late apoptotic (AnV+DAPI+) neutrophils is shown. B) BMNs were isolated from Mrp8:Metfl/fl and control Mrp8;Metwt/wt littermates and exposed to L. mexicana-DsRed for 2 or 20h at indicated MOI. The frequency of live (DAPI-) BMNs was assessed by flow cytometry. Data are representative of 3 independent experiments with n≥ 2. (TIF) [file ppat.1010247.s001.tif]

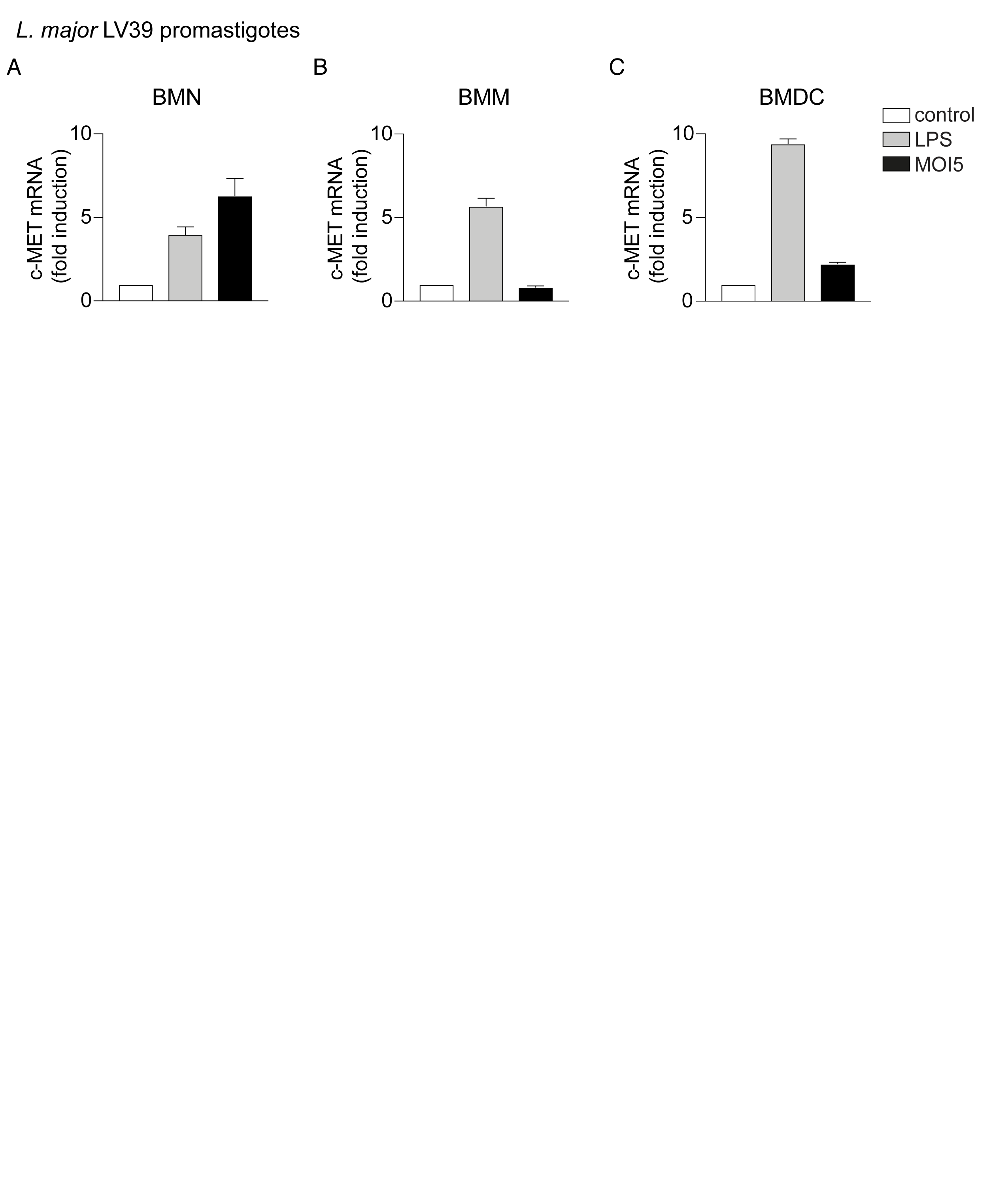

Supplement: S2 Fig — A) BM neutrophils (BMNs) were isolated and co-cultured in vitro with metacyclic L. major LV39 for 16h at a MOI of 5. c-MET mRNA levels were analysed by RT-qPCR. Unexposed BMNs were used as negative control and LPS treated cells as positive control. B) BM-derived macrophages (BMMs) and C) BM-derived dendritic cells (BMDCs) were similarly infected and c-MET expression was assessed by RT-qPCR. Data are normalized to endogenous levels of HPRT mRNA and expressed as fold increase relative to expression levels measured in control cells. Data are representative of ≥ 3 experiments, n = 3/group. (TIF) [file ppat.1010247.s002.tif]

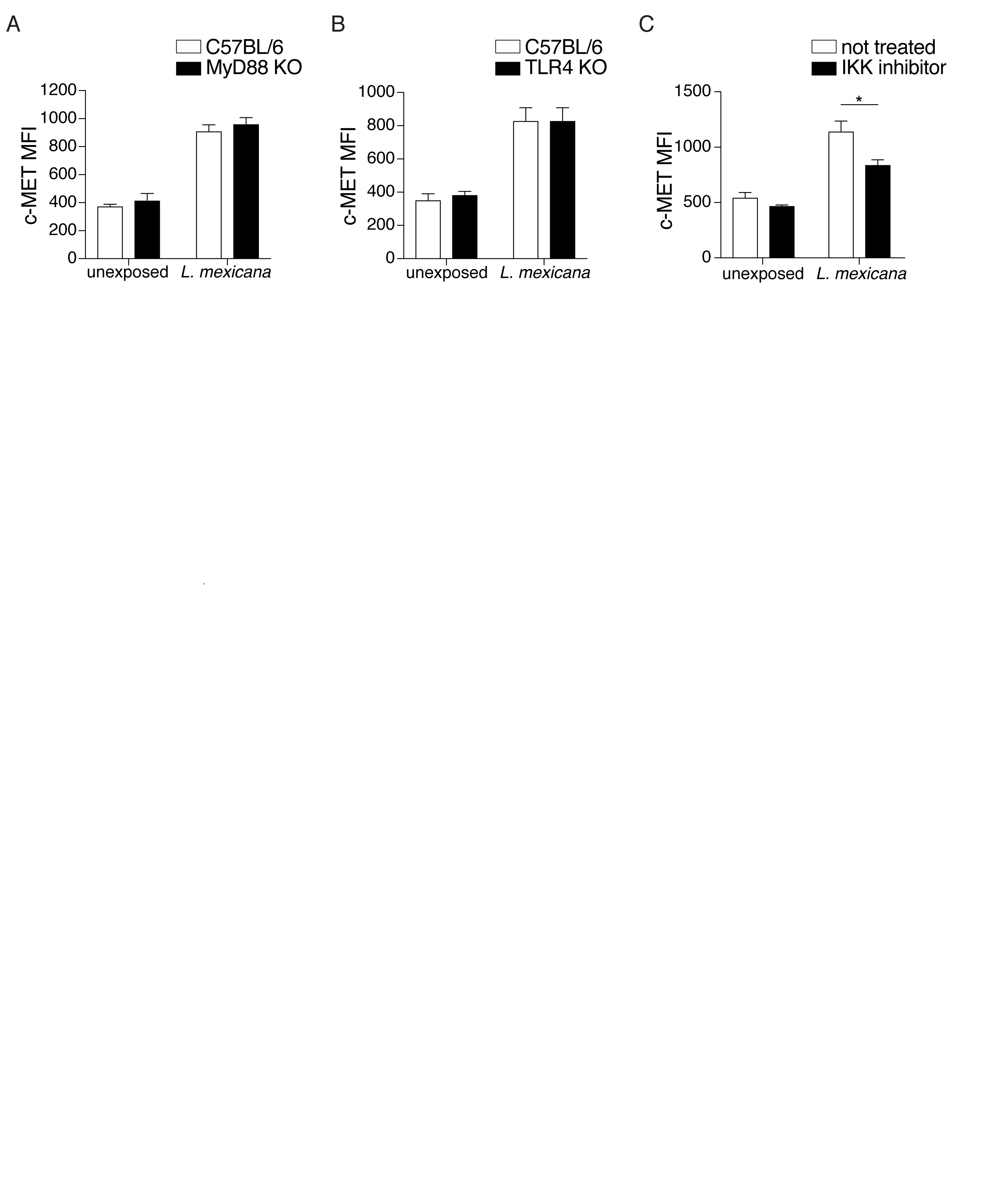

Supplement: S3 Fig — A) BMNs were isolated from MyD88-/- and C57BL/6 mice and co-cultured for 16h with L. mexicana metacyclic promastigotes at a MOI of 5. c-MET expression in infected or non-infected (unexposed) neutrophils was assessed by flow cytometry. The MFI of a representative experiment is shown. B) BMNs isolated from TLR4-/- and C57BL/6 mice were processed as indicated in A. The MFI of a representative experiment is shown. C) BMN were isolated from C57BL/6 mice and pre-treated for 1h with the IKK inhibitor III. Cells were then exposed to L. mexicana for 16h. c-MET expression was assessed by flow cytometry. The representative MFI is shown. Data are representative of ≥ 2 experiments, n≥3. (TIF) [file ppat.1010247.s003.tif]

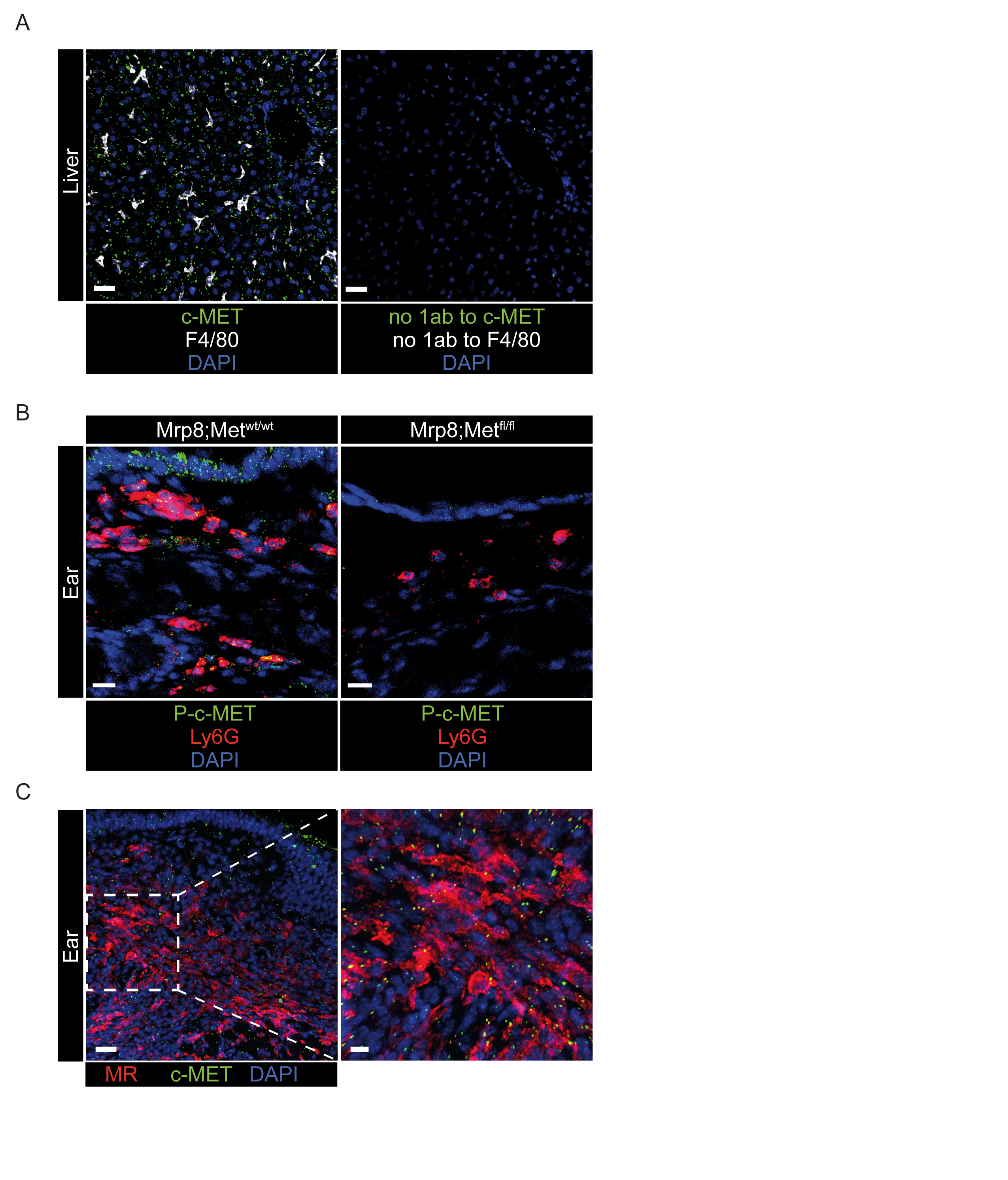

Supplement: S4 Fig — A) Representative histology pictures of naïve liver stained with c-MET (green), F4/80 (white) and DAPI (blue) on the left. Liver stained in absence of the first step c-MET and F4/80 antibodies on the right, as control for primary antibodies specificity. Scale bar: 30μm. B) Representative pictures of Mrp8;Metwt/wt and Mrp8;Metfl/fl ears 6 hours p.i with 106 L. mexicana, staining for phospho-c-MET (P-c-MET, green), Ly6G (red) and DAPI (blue). Scale bars: 30μm. Note the absence of P-c-MET staining in neutrophils in Mrp8;Metfl/fl ears. Scale bar: 10μm. C) Representative histology picture of six weeks infected ear, showing immunofluorescent staining for MR+ dermal macrophages (red), c-MET (green) and DAPI+ nuclei (blue). Scale bar: 30μm (left), enlargement of the defined area (right), scale bars: 10μm. Data are representative of ≥1 experiments. (TIF) [file ppat.1010247.s004.tif]

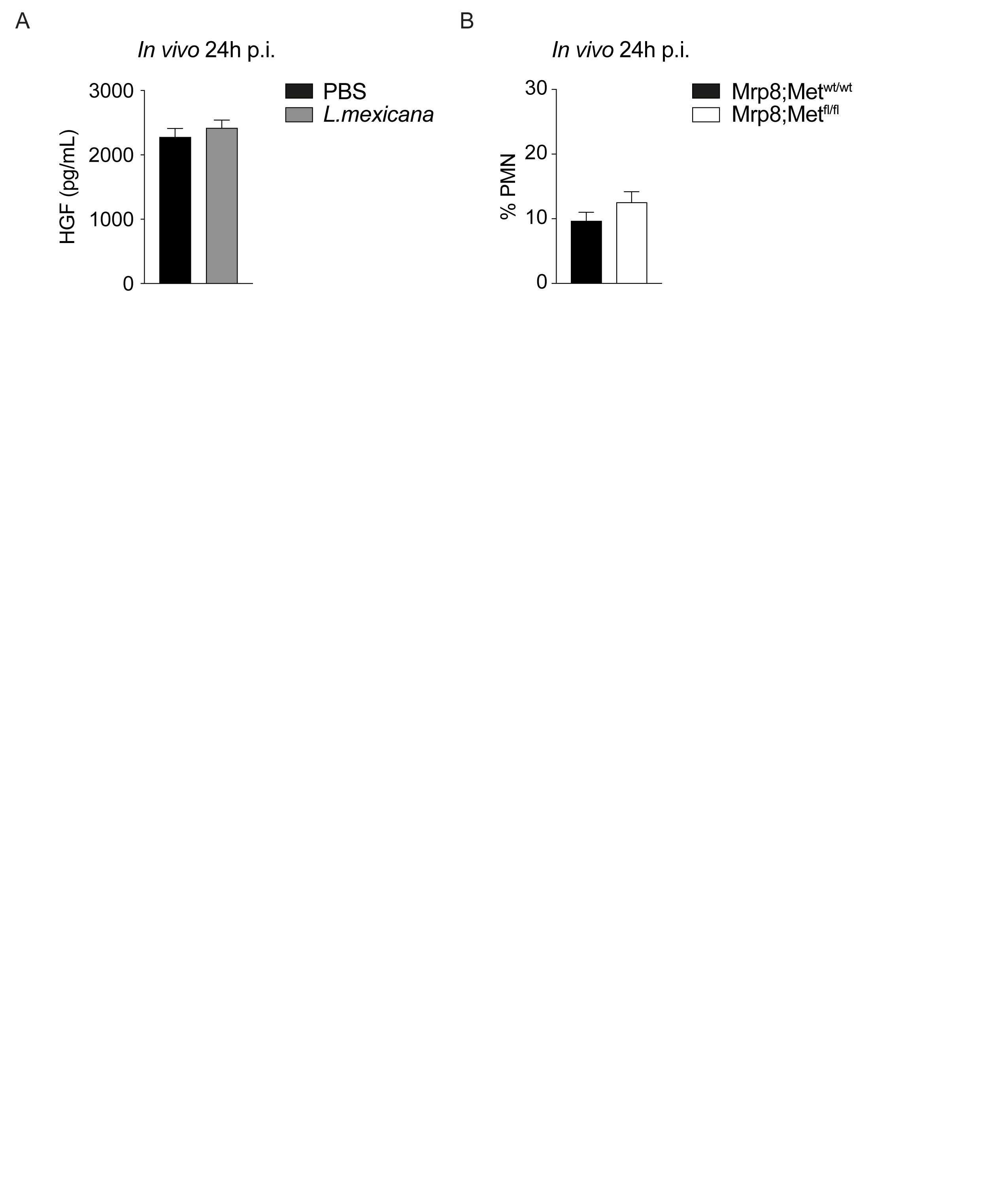

Supplement: S5 Fig — A) C57BL/6 mice were infected i.d. with 106 L. mexicana metacyclic promastigotes. Twenty-four hours late, the infected ears were collected and homogenized in RIPA buffer using a tissue lyser and the level of HGF analysed by ELISA. PBS-injected ears were used as a control. B) Mrp8;Metfl/fl mice deficient for c-MET in PMNs and Mrp8;Metwt/wt control littermate were infected with 106 L. mexicana metacyclic promastigotes in the ear dermis. Twenty-four hours post infection, ears were collected and digested to obtain a cell suspension. The frequency of CD45+CD11b+Ly6G+ PMNs was assessed by flow cytometry. Data are representative of ≥2 independent experiments, n≥5/group. (TIF) [file ppat.1010247.s005.tif]
